# Implementation and Assessment of a Novel Telehealth Education Curriculum for Undergraduate Medical Students

**DOI:** 10.30476/jamp.2021.89447.1375

**Published:** 2021-07

**Authors:** JASMIN MAHABAMUNUGE, LAUREN FARMER, JOANNA PESSOLANO, NISHA LAKHI

**Affiliations:** 1 New York Medical College, Department of Obstetrics and Gynecology, Valhalla, NY, USA; 2 Duke University School of Medicine, Department of Obstetrics and Gynecology, Durham, NC, USA; 3 Richmond University Medical Center, Department of Obstetrics and Gynecology, Staten Island, NY, USA

**Keywords:** Telehealth, COVID-19, Medical education, Virtual

## Abstract

**Introduction:**

Despite its healthcare advantages and expanded use during the COVID-19 pandemic, telehealth is not included in many medical school curricula.

**Methods:**

In this prospective mixed methods study (n=52), we created a novel Telehealth Education Curriculum (TEC) for the third year Obstetrics and Gynecology (Ob/Gyn) rotation at New York Medical College during COVID-19. The TEC included supervised telehealth patient encounters via video conference [and a virtual Objective Structured Clinical Encounter (vOSCE)] designed to simulate a telehealth encounter (Zoom Video Communications, Inc.). We measured student perceptions of the TEC via two 4-point Likert surveys, which included free response questions, administered via SurveyMonkey between April and June 2020. Participation was voluntary and responses were de-identified. We computed means and response distributions across survey questions using SPSS; IBM version 19.

**Results:**

The response rate was 92% for both the Telehealth (33/36) and vOSCE (48/52) surveys. Seventy-six percent (25/33) strongly or moderately agreed that telehealth and in-person patient encounters have similar educational value. Eighty-three percent (40/48) strongly or moderately agreed the vOSCE provided a valuable patient interaction. Ninety-seven percent (32/33) strongly or moderately agreed the telehealth encounters should continue during COVID-19 restrictions versus 82% (27/33) agreeing they should be incorporated into the curriculumpost COVID-19.

**Conclusion:**

Almost all students responded that the TEC should continue during COVID-19 and most agreed it should be incorporated into the Ob/Gyn clerkship permanently, after COVID-19. We found vOSCEs to be an effective method for teaching telehealth to medical students. Key challenges identified by students included adjusting to a virtual format, lack of body language, and communicating empathy virtually. Positive takeaways included practice with telemedicine and an opportunity for continued clinical education during COVID-19.

## Introduction

Telemedicine or “Telehealth” has grown exponentially in the U.S. over the past few decades and current trends in the healthcare environment fuel this expansion into the future ( 1 , 2 ). Telehealth involves the use of telecommunications and virtual technology to deliver healthcare outside of the traditional brick and mortar healthcare facility. This platform has been shown to reduce inefficiencies in the delivery of healthcare by improving access for underserved and rural populations ( 3 , 4 ). Telehealth has also been shown to be cost-effective, improve health outcomes, and enhance patient social support ( 5- 8). Kansas, is one of the longest running practices of its kind worldwide. The practice began in 1995 and connected an oncologist at KUMC with a rural medical center in Hays, Kansas. Fifteen years later, the practice continues to thrive at Hays Medical Center and has also expanded to include two additional sites within the state-the Northeast Kansas Center for Health and Wellness in Horton and Goodland Regional Medical Center in Goodland-that offer regularly scheduled teleoncology clinics. While the KUMC practice has witnessed an expansion in service sites throughout its history, the practice has seen a significant decrease in the costs associated with providing such services since its inception. The cost decrease can, in part, be attributed to an increase in the number of teleoncology visits conducted through the practice since it began (In Fiscal Year 1995). Recently, telehealth has also proven to be rather dynamic as it has been applied in both primary care settings and for specialty consultations ( 9 ).

Despite the multiple innovative applications of digital technology in healthcare, telehealth is not readily adopted by all healthcare providers and many medical schools still do not include telehealth in their curricula ( 2 , 10 ). Additionally, the American Medical Association (AMA) recommends telehealth training of medical students and residents. However, a recent survey by the Association of American Medical Colleges (AAMC) found that only 58% of medical schools currently provide telehealth training ( 11 , 12 ).

Due to the new challenges faced by medical schools during the COVID-19 pandemic, novel curricula must be considered ( 13 , 14 ). Medical schools have been challenged with adapting and innovating new curricula aimed at creating clinical experiences for students that are remote from the hospital ( 15 ). To ensure the delivery of quality clinical education despite the pandemic, we created a Telehealth Education Curriculum (TEC) for the Obstetrics and Gynecology (Ob/Gyn) third year clinical rotation at New York Medical College, which was implemented in April 2020. The novel TEC included medical student telehealth encounters with real patients and a virtual OSCE (vOSCE) with standardized patients to resemble a telehealth encounter. We chose to incorporate an OSCE into the TEC curriculum as they have been shown to improve clinical skills in educational settings ( 16 ). Students were asked to complete surveys on their perceptions of the TEC and responses were used to assess its educational value.

The objective of this study was to measure student perceptions of a novel Telehealth Education Curriculum for the virtual obstetrics and gynecology core clerkship during the COVID-19 pandemic and evaluate best practices for future telehealth curriculum implementation. A secondary objective was to contribute to current medical education literature on telehealth curricula, and their utility, to continue finding new innovations in medical student education during the COVID-19 pandemic and beyond.

## Methods

### Study Design/ Participants

This prospective mixed methods study of 52 medical students was conducted at New York Medical College School of Medicine (NYMC SOM), located in Valhalla, New York. It is one of the nation’s oldest private health sciences universities (est. 1860) and provides a comprehensive educational program with the goal of developing well-rounded medical students who will become resilient, compassionate, and skilled physicians. The SOM is proud of its strong foundational science education, diverse affiliated clinical training sites, and the commitment of its faculty and administration to medical student education.

In light of the COVID-19 pandemic, which provided a significant barrier to undergraduate medical education, and specifically clinical education, the third year Obstetrics and Gynecology (Ob/Gyn) medical student core clerkship was converted to a four-week virtual format in April 2020. The students originally scheduled to complete the standard Ob/Gyn clerkship in April and May 2020 were automatically enrolled in the virtual Ob/Gyn clerkship. This put the onus on faculty to rapidly develop and implement a new virtual curriculum that allowed for clinical learning and interaction in this new digital era. This new curriculum featured didactic sessions, case-based presentations, and interactive learning workshops via Zoom. Didactic activities included presentation of video-based surgical cases, interactive board question review, and discussion of general obstetrics and gynecology topics. Additionally, a novel Telehealth Education Curriculum (TEC) consisting of virtual Observed Structured Clinical Examinations and actual telehealth patient encounters was implemented as part of the virtual Ob/Gyn third year clerkship. We evaluated the student perceptions of the novel TEC following their successful completion of the virtual Ob/Gyn clerkship.

To be considered eligible to participate in the study, the students were required to be in their third year at NYMC SOM, enrolled in the virtual Ob/Gyn clerkship, and participating in the virtual Ob/Gyn clerkship and TEC. Students were provided with explicit information explaining that their participation in surveys was voluntary and their responses would be utilized for research purposes only. They were also informed that responses would be de-identified and accessed only by the research team data handlers with no impact on their grading. Two authors, JM and LF, were responsible for all data handling and were not involved in any aspect of medical student grading for the clerkship. NL and JP were able to access student data after it was processed and fully de-identified without the ability to re-identify students given their roles at the School of Medicine. All study procedures were approved by and compliant with the New York Medical College Institutional Review Board (protocol #14323).

### Novel Telehealth Education Curriculum (TEC) Design and Assessment

The novel Telehealth Education Curriculum (TEC) consisted of two components: the telehealth encounter and the virtual Observed Structured Clinical Examination (vOSCE). For the telehealth encounter, each student completed at least one telehealth visit via a 3-way video conference call with an Ob/Gyn patient and attending physician, before the end of the 4-week virtual clerkship. Following the telehealth visit, students completed a clinical note in the Subjective, Objective, Assessment, and Plan (SOAP) note structure and a survey entitled Telehealth Evaluation for 3^rd^ year Ob/Gyn Clerkship. Students then virtually met with their attendings for a telehealth debrief in which verbal and written feedback was provided.

The second component of the novel TEC was the virtual OSCE designed to mimic a telehealth patient encounter, conducted via Zoom Video Communications, Inc. The blueprint for the vOSCE included completion of a standardized patient encounter with a physical exam and history (20 minutes), a post-encounter note that included a differential diagnoses, assessment, and plan (15 minutes), followed by two debriefing sessions with faculty focused on communication skills (15 minutes) and clinical reasoning (40 minutes). Students also had the opportunity to collaborate with another student who had independently completed the same case, while individually writing their assessment. The standardized patients presented with a common obstetric and gynecologic complaint (i.e. primary amenorrhea, postmenopausal bleeding, antenatal visit). Faculty observed the vOSCEs, reviewed post-encounter assessments, and evaluated students using the following criteria: medical knowledge, interpersonal skills and communication, empathy, and development of a differential diagnoses. Following the vOSCE, students completed the Virtual OSCE Evaluation Form for the 3rd year Ob/Gyn clerkship to provide feedback about their experience with the vOSCE and its value as an educational tool.

### Data Collection

To assess student perceptions of the novel TEC curriculum, students in the first two cohorts of the virtual Ob/Gyn third year clerkship were asked to complete two 4-point Likert surveys consisting of nine student perception questions and four free response questions. Possible answer choices for the Likert survey questions were as follows:1=Strongly agree, 2=Moderately agree, 3=Moderately disagree, 4=Strongly disagree. Free response questions for both surveys asked the following: 1) What was most successful about the session? 2) What was most challenging about the session? 3) What were my top takeaways from this session (clinical and communication skills based)? 4) Any Additional comments or suggestions for improvements?

The two surveys were distributed to students at the conclusion of their virtual Ob/Gyn clerkship. One survey corresponded to the telehealth encounter and the other to the virtual OSCE. Responses were de-identified upon completion and stored in a secure database.

### Analysis

We conducted all analyses using the Statistical Package for the Social Sciences (SPSS; IBM version 19). Likert data were analyzed by computing means, and response distributions, for each question to compare self-reported student agreement with survey statements.

For qualitative analysis of free response questions, the research team reviewed all qualitative data individually to identify salient themes. The research team then met as a group to resolve discrepancies in interpretations and reach final theme interpretation. Qualitative data was reviewed until no new themes emerged from the analysis. Illustrative quotes were collected to represent all salient themes. 

## Results

A total of 52 students participated in the virtual Ob/Gyn clerkship with 25 in the first cohort and 27 in the second. The telehealth encounter component of the TEC was still being piloted with the first cohort of students, so their participation in the telehealth encounter was optional. Based on faculty availability and student interest, 9 out of the 25 students in the first cohort completed the telehealth encounter. However, all 25 participated in the vOSCE because it was not optional. All 27 students in the second cohort completed both components of the TEC: the telehealth encounter and vOCSE. Of the 52 students who participated in the first two cohorts of the virtual Ob/Gyn clerkship, 36 completed the Telehealth encounter and 52 completed the vOSCE. Of these students, 33 completed the Telehealth survey (92%) and 48 completed the vOSCE survey (92%) (Tables 1 and 2).

**Table 1:** Likert Survey Medical Student Perceptions of the novel TEC: Telehealth Encounter. N=33

Telehealth Evaluation Survey Statements	Strongly Agree	Moderately Agree	Moderately Disagree	Strongly Disagree
The physician instructor explained medical history and other pertinent facts prior to the Telehealth patient encounter.	28 (84.9%)	2 (12.1%)	1 (3.0%)	0 (0%)
This Telehealth experience provided me with a valuable patient interaction.	21 (63.4%)	11 (33.3%)	1 (3.0%)	0 (0%)
The content of the physician debriefing played a useful role in my clinical skills training.	24 (72.3%)	9 (27.3%)	0 (0%)	0 (0%)
I felt that it was a good use of my time to participate in a Telehealth encounter.	25 (75.8%)	8 (24.2%)	0 (0%)	0 (0%)
The telehealth activity helped develop practical skills for effective communication with patients.	22 (66.7%)	8 (24.2%)	3 (9.1%)	0 (0%)
This session increased my confidence in interviewing a patient presenting with a gynecological complaint.	22 (66.7%)	9 (27.3%)	2 (6.1%)	0 (0%)
The educational value of a telehealth patient interview was similar to an in-person patient interview.	11 (33.3%)	14 (42.4%)	7 (21.2%)	1(3.0%)
Telehealth encounters/education should be part of the medical student clerkships during social distancing secondary to COVID-19 restrictions.	26 (78.8%)	6 (18.2%)	1 (3.0%)	0 (0%)
Telehealth encounters/education should be part medical student clerkships going forward (post COVID-19).	14 (42.4%)	13 (39.4%)	6 (18.2%)	0 (0%)

TEC: Telehealth Education Curriculum; vOSCE: virtual Objective Structured Clinical Encounter

**Table 2:** Likert Survey Medical Student Perceptions of the novel TEC: vOSCE. N=48

vOSCE Evaluation Survey Statements	Strongly Agree	Moderately Agree	Moderately Disagree	Strongly Disagree
This virtual SP experience provided me with valuable patient interaction.	22 (45.8%)	18 (37.5%)	2 (4.2%)	6 (12.5%)
The scenario content was relevant to my clerkship.	33 (68.8%)	9 (18.8%)	0 (0%)	6 (12.5%)
My interaction with the SP was appropriate and realistic.	18 (37.5%)	21 (43.8%)	6 (12.5%)	3 (6.3%)
Collaborating with a colleague while writing the post-station note enhanced the virtual OSCE experience.	23 (47.9%)	8 (16.7%)	15 (31.3%)	2 (4.2%)
The content of the debriefing played a useful role in my clinical skills training.	20 (41.7%)	18 (37.5%)	6 (12.5%)	4 (8.3%)
This session increased my confidence in interviewing a patient presenting with a gynecological complaint.	15 (31.3%)	23 (47.9%)	5 (10.4%)	5 (10.4%)
This session increased my confidence in navigating a patient interview using a virtual platform.	23 (47.9%)	20 (41.7%)	1 (2.1%)	4 (8.3%)
A virtual-OSCEs is a valuable tool to train students in Telemedicine.	21 (43.8%)	19 (39.6%)	4 (8.3%)	4 (8.3%)
Virtual-OSCEs should be part of 3rd year medical student clerkships going forward.	12 (25%)	19 (39.6%)	13 (27.1%)	4 (8.3%)

TEC: Telehealth Education Curriculum; vOSCE: virtual Objective Structured Clinical Encounter

Student responses to the Telehealth survey revealed that 76% strongly or moderately agreed that the educational value of a telehealth patient interview was similar to an in-person patient interview. Additionally, 97% strongly or moderately agreed that their telehealth encounter provided them with a valuable patient interaction. When asked if the telehealth encounter should continue during the COVID-19 pandemic, 97% strongly or moderately agreed. However, when asked if the telehealth encounter should be incorporated into the Ob/Gyn clerkship curriculum permanently, when the pandemic comes to an end, 82% strongly or moderately agreed.

Student answers to free response questions on the Telehealth survey revealed key themes for the first three out of four questions. When asked “What was most successful about the session?” students mostly reported: getting virtual clinical exposure to “real patients” during a pandemic, practicing Ob/Gyn history taking, and being taught by knowledgeable attendings who helped develop clinical reasoning skills. When asked “What was most challenging about the session?” most students identified: technical challenges, inability to physically examine the patient, and difficulty communicating nonverbally. When asked “What were my top takeaways from this session?” most students reported: telehealth visits are similar to in person visits, telehealth visits are a valuable tool and save time for patients, communication skills are important in a virtual format, and time management is important when acquiring patient histories virtually (Table 3).

**Table 3:** Telehealth survey free response questions and identified student answer themes.

Questions and identified response themes	Selected quotes
**1. What was most successful about the session?**	
Getting virtual clinical exposure to “real patients” during a pandemic.	“Having an opportunity to interact with patients regardless of being on a 'virtual' rotation”.
	“Being able to learn more and interview a real patient. Having that actual patient exposure is one of the most important things about M3”.
	“Speaking with true patients (not SPs) is always beneficial and Ob/Gyn was one of the only rotations to arrange this, so that was a major plus”.
2. Practicing Ob/Gyn history taking.	“Being able to get the history of an obgyn patient and present to my attending was useful and helped me feel like I was getting clinical exposure”.
3. Being taught by knowledgeable attendings who helped develop clinical reasoning skills.	“The 2 physicians I was paired with were both extremely helpful and provided a lot of information about the encounter. Both answered my questions thoroughly and what to expect during the encounter. I thought it was a good experience”.
	“Direct feedback from the provider was very helpful”.
	“The opportunity to work directly with attending physicians and answer their questions related to the case”.
**What was most challenging about the session?**	
1. Technical challenges	“Technological issues with 3-way calling”.
	“The technology sometimes didn’t always work”.
2. Inability to physically examine the patient.	“With OB/Gyn telemedicine visits it seems difficult to capture a complete picture of a patient at times because for one, you are not able to perform a pelvic exam”.
	“Not being able to physically examine the patient”.
3. Difficulty communicating with nonverbally.	“Not really being able to engage how the patient was doing based on the physical cues”.
	“Not being able to play off the patient’s expressions and emotions”.
**What were my top takeaways from this session?**	
1. Telehealth visits are similar to in person visits.	“They helped solidify clinical information similarly to how in-person encounters do. It was also beneficial for practicing communication skills”.
	“That virtual interviews are not too different in terms of communications and personal skills, and history taking”.
2. Telehealth visits are a valuable tool and save time for patients.	“Telehealth is a huge time saver for the patient and also allows them to ask any immediate questions they have and check in more often”.
	“That telehealth visits can still be useful and productive, and are a valuable tool”.
3. Communication skills are important in a virtual format.	“Communication skills even more important in this format”.
4. Time management is important when acquiring patient histories virtually.	“How to gather a focused history without seeing the patient in person”.

Medical student responses to the vOSCE survey revealed that 83% strongly or moderately agreed the vOSCE standardized patient experience provided a valuable patient interaction. When asked if the vOSCE session increased their confidence navigating a patient interview using a virtual platform, 90% of students strongly or moderately agreed. Additionally, 83% strongly or moderately agreed that the vOSCE is a valuable tool to train students in telemedicine. Finally, 65% of respondents strongly or moderately agreed the vOSCEs should be part of the Ob/Gyn clerkship going forward, including after the COVID-19 pandemic.

Student answers to free response questions on the vOSCE survey again revealed key themes for the first three out of four questions. When asked “What was most successful about the vOSCE session?” students mostly reported: increased comfort and familiarity with the virtual encounter format, opportunity to experience Ob/Gyn patient encounters during COVID-19, and collaboration with peers. When asked “What was most challenging about the session?” students identified: technical difficulties/adjusting to a virtual format, the virtual physical exam, and difficulty connecting with patients given limited ability to read body language and communicate nonverbally. When asked “What were my top takeaways from this session?” students mostly reported: the vOSCE was helpful for practicing Ob/Gyn history taking and developing a differential diagnosis, ability to experience and practice using telemedicine, telemedicine is a good tool for student learning, and practice with nonverbal communication (Table 4).

**Table 4:** vOSCE survey free response questions and identified student answer themes.

Questions and identified response themes	Selected quotes
**What was most successful about the session?**
1. Increased comfort and familiarity with the virtual encounter format.	“Having to interact with the standardized patient virtually and feeling comfortable as the session went on”.
	“Familiarizing with the first-time awkward feeling of speaking with patients through a screen”.
	“Getting comfortable with the format”.
2. Opportunity to experience Ob/Gyn patient encounters during COVID-19.	“To be able to interview a patient with a gyn complaint since we weren’t able to do so in person”.
	“It provided me with the ability to practice history taking in a gyn encounter, which I hadn't had the entire rotation”.
3. Collaboration with peers.	“Having the chance to do a virtual interview and having another student to discuss the note with”.
	“I feel like getting to work in tandem with someone else and see how they apply clinical reasoning skills helped me to improve my own note-writing and differential diagnosis skills”.
**What was most challenging about the session?**	
1. Technical difficulties/adjusting to a virtual format.	“Technical difficulties and lag”.
	“I had some internet issues initially, definitely flustered me a bit”.
2. The virtual physical exam.	“Having to do the physical examination by asking questions was a bit hard to navigate”.
	“Asking questions about the components of the physical exam, as opposed to doing the actual maneuvers”.
3. Difficulty connecting with patients given limited ability to read body language and communicate nonverbally.	“Not being able to express appropriate empathy or read the patient's body language”.
	“Difficult to adjust to being on a virtual platform in terms of showing empathy and utilizing communication skills”.
	“Not having the body language as part of communication”.
**What were my top takeaways from this session?**
1. The vOSCE was helpful for practicing Ob/Gyn history taking and developing a differential diagnosis.	“Very helpful to have some communication with a ‘patient’ during the course, given the circumstances”.
2. Ability to experience and practice using telemedicine.	“Greater comfort with telemedicine encounters”.
	“It was a really nice experience even though it’s new and nerve racking at first”.
3. Telemedicine is good tool for student learning.	“Telemedicine SPs are an effective way to teach students and can be very helpful”.
	“Telemedicine is an important tool in medicine”.
Practice with nonverbal communication.	“Rapport building and demonstrating empathy must be even more intentional on a virtual platform because subtleties can be missed”.
	“There is a different way of expressing empathy in these interactions”.
	“Communication is especially important when interactions are virtual”.

TEC: Telehealth Education Curriculum; vOSCE: virtual Objective Structured Clinical Encounter

## Discussion

When the COVID-19 pandemic hit the Tristate Area and students were dismissed from in person clinical rotations, faculty and student researchers in the Department of Obstetrics & Gynecology at New York Medical College drafted and implemented a virtual Ob/Gyn clerkship and novel Telehealth Education Curriculum (TEC) in a matter of weeks. After the first two cohorts of students completed the virtual Ob/Gyn clerkship and novel TEC, we assessed student perceptions of the TEC. In general, most students agreed that the telemedicine encounters provided similar educational opportunities as in-person encounters and were a valuable clinical tool that should be incorporated into future Ob/Gyn clerkship curricula, during and after the pandemic. Furthermore, student responses were both positive and thankful for the chance to continue their learning and experience using an emerging healthcare platform in the midst of a rapidly changing learning environment. While students indicated initial difficulty adjusting to a virtual format and communicating with fewer nonverbal cues, most ultimately expressed increased comfort using telehealth after completing the TEC.

The information presented in this study including the tools used to implement the curriculum and format by which student perceptions were assessed may be utilized by other institutions interested in developing a telehealth curriculum for their students. While some US medical schools have created virtual student rotations, or exams, and proven them effective for clinical learning, few have incorporated focused teaching on the practice of telehealth itself ( 17- 19). Proceeding with assessment to determine competency for graduation from medical school, and maintaining performance standards for graduating doctors is an unprecedented challenge under pandemic conditions. This challenge is hitherto uncharted territory for medical schools and there is scant guidance for medical educators. In early March 2020, Duke-National University Singapore Medical School embraced the challenge for ensuring competent final year medical students could complete their final year of studies and graduate on time, to enter the medical workforce in Singapore without delay. This paper provides details of how the final year clinical performance examinations were planned and conducted during the COVID-19 pandemic. The aim of the paper is to provide guidance to other medical schools in similar circumstances who need to plan and make suitable adjustments to clinical skills examinations under current pandemic conditions. The paper illustrates how it is possible to design and implement clinical skills examinations. Our study findings are consistent with the few other studies reporting on medical student telehealth education including increased student comfort using telehealth following the curriculum and student agreement about the value of telehealth education ( 20 , 21 ). Patient interest in and satisfaction with telehealth are high. No comprehensive U.S. undergraduate medical education curriculum teaching telehealth University of the Health Sciences (USU). Other studies also reveal initial student discomfort using telehealth platforms, student-reported technological issues, and student agreement that telehealth provides similar learning opportunities compared to in-person encounters ( 20 - 22 ). Interestingly, our students identified challenges communicating with patients nonverbally and expressing empathy via a virtual platform: “rapport building and demonstrating empathy must be even more intentional on a virtual platform because subtleties can be missed.” However, with instruction and continued practice, students were able to overcome this obstacle,  and learned “there is a different way of expressing empathy in these interactions and there’s never not an opportunity to show empathy and compassion to a patient. ” The need for effective communication adapted for the virtual healthcare setting has been recognized but further studies in the clinical setting are necessary ( 23 , 24 ). Lastly, to our knowledge, our study is the first to evaluate medical student desire for telehealth training during and after the COVID-19 pandemic. Thus, the present study underscores the importance of training future physicians to use this ever evolving and increasingly popular medical technology.

In recent years, telehealth has been increasingly employed to deliver quality healthcare to diverse patient groups ( 2 , 8 ). Pre-COVID-19 pandemic trends supported its continuous growth but the current health crisis has accelerated the rate at which healthcare facilities are adopting and utilizing telehealth across practice sites ( 25 , 26 ). This rapid integration is unlikely to be a fad. Evidence suggests that following the pandemic, telehealth will prevail given its copious benefits for patients and providers alike ( 27- 29). Because telehealth is here to stay as another healthcare delivery modality, it is important to train future providers to successfully use this interface ( 10 , 30 ). In fact, many medical students are already expressing interest in telehealth education and have plans to use telehealth in their future careers as physicians ( 20 , 31 , 32 ). By delivering telehealth training to medical student, our novel TEC provides an opportunity to bridge a much-anticipated knowledge gap between future physicians and medical technologies they will be expected to know and use. Overall, the COVID-19 pandemic provided an educational incentive to address the already existing need for medical student telehealth education through innovative educational curricula.

## Conclusion

Our data strongly support an urgent need for current and future medical student telehealth education curricula. Still, our study presents with some limitations including a modest sample size. Additionally, we solely evaluated medical students enrolled in the third-year obstetrics and gynecology rotation during the COVID-19 pandemic at a single institution. Therefore, more studies evaluating medical student perceptions of telehealth education curricula are needed to examine if our findings and conclusions can be extrapolated to other fields of medical education. Student free responses indicate areas for continued improvement when implementing telehealth curricula including a demand for more telehealth encounters, more vOSCE cases and virtual SP feedback, and follow up telehealth visits. Other than these area of improvement, medical students indicated overall great satisfaction with our novel TEC.

Given how well received our TEC was at New York Medical College during the COVID-19 pandemic and that students expressed interest in continued telehealth learning, we plan to incorporate the TEC into future clerkship curricula. Currently, the third year Ob/Gyn clerkship is split between virtual and in-person learning. We plan to integrate student feedback from this pilot project by including more telehealth encounters and modify/adapt the curriculum according to emerging student needs and healthcare trends as appropriate. 

## Funding/Support:

The authors received no specific funding for this work.
